# Cardiac Magnetic Resonance Guidance for the Pathogenetic Definition of Cardiomyopathies

**DOI:** 10.1007/s11886-025-02233-8

**Published:** 2025-04-16

**Authors:** Bishow Paudel, Jonathan Pan, Cristiane C. Singulane, Shuo Wang, Matthew Thomas, Michael Ayers, Steven Philips, Amit R. Patel

**Affiliations:** Department of Medicine, Division of Cardiovascular Medicine, University of Virginia, Charlottesville, VA USA

**Keywords:** Genetic/Inherited cardiomyopathy, Cardiac Magnetic Resonance (CMR) Features, CMR-pathogenetic, Genes, Inflammation

## Abstract

**Purpose of Review:**

Pathogenetics is the study of genetics in disease pathogenesis. Many abnormal gene alleles have been identified in cardiomyopathies, but their clinical utility remains limited. This review aims to examine the integration of cardiac MRI (CMR) with genetic data to enhance early detection, prognostication, and treatment strategies for cardiomyopathies.

**Recent Findings:**

CMR is the gold standard imaging modality for cardiomyopathy evaluation, capable of detecting subtle structural and functional changes throughout the disease course. When applied to patients with genetic mutations, with or without phenotypic expression, CMR aids in early diagnosis and risk stratification. Cardiomyopathies can be categorized into at least seven clinical groups based on morphology, function, and genetic associations: (1) Dilated cardiomyopathy (DCM), (2) Hypertrophic cardiomyopathy (HCM), (3) Restrictive cardiomyopathy, including transthyretin amyloidosis (ATTR-CM), iron overload, and Anderson-Fabry disease, (4) Arrhythmogenic cardiomyopathy (ACM), (5) Non-dilated left ventricular cardiomyopathy (NDLVC), (6) Peripartum cardiomyopathy, and (7) Muscular dystrophy-related cardiomyopathy. We have described left ventricular noncompaction (LVNC) as a morphological trait rather than a distinct cardiomyopathy. Emerging CMR and genetic data suggest an inflammatory component in DCM and ACM, with potential therapeutic implications for immunotherapy. Advanced CMR techniques, such as quantitative perfusion, can distinguish cardiomyopathies from ischemic heart disease and detect early microvascular dysfunction, particularly in ATTR-CM and HCM. Late gadolinium enhancement (LGE) and parametric mapping (T1 and extracellular volume [ECV]) further enhance early diagnosis, prognostication and treatment response by assessing fibrosis and myocardial composition.

**Summary:**

The integration of CMR and genetic insights improves our understanding of cardiomyopathy pathogenesis, aiding in early diagnosis and prognostic assessment. Future research should leverage artificial intelligence (AI) to analyze genetic and radiomic CMR features, including perfusion data, to establish a comprehensive pathogenetic framework. This approach could refine disease classification, identify novel therapeutic targets, and advance precision medicine in cardiomyopathy management.

## Introduction

Pathogenetics is the study of mechanisms in which genetic abnormalities result in disease phenotypes. There are hundreds of pathogenic mutations associated with cardiomyopathies. As a result, the connection between these molecular defects, the pathogenesis of disease, and the manifestation of symptoms is often poorly understood. Deciphering the pathogenetic variations can be challenging, requiring a deep understanding of the metabolic and molecular pathways that contribute to distinct disease phenotypes and clinical presentations. Cardiac magnetic resonance (CMR) is a powerful imaging modality that can be used to characterize abnormal myocardial tissue in those with suspected genetic cardiomyopathies. With its high spatial and temporal resolution, CMR is considered the gold standard for quantifying cardiac chamber size, mass, and function with high accuracy and reproducibility [1, 2]. In this article, we reviewed the role of CMR in delineating genetic phenotypes and elucidating the pathogenesis of different cardiomyopathies.

## Genetics and Cardiology

The heart is comprised of different cell types such as endothelial cells, vascular smooth muscle cells, fibroblasts, pericytes, immune-related cells, and cardiomyocytes. There are multiple molecular sites in the heart in which genetic mutations can disrupt the electrical and mechanical function. The sarcomere, the fundamental unit of contraction, consists of thick and thin filaments that slide over each other and are linked at intercalated disks for coordinated action. The cardiomyocyte membrane contains transverse tubules that facilitate rapid action potential transmission, leading to calcium influx and muscle contraction. To meet their high energy demands, cardiomyocytes are rich in mitochondria and have a supportive cytoskeletal network. Mutations in either the structural or regulatory proteins can cause a heterogenous expression of cardiomyopathy [3]. This can be difficult to detect when there is concomitant non-genetic heart disease.

Multiple societies including the Heart Failure Society of America and the American College of Medical Genetics recommend a systematic approach to genetic evaluation of cardiomyopathies [4, 5]. A detailed family history of three generations should be documented in a pedigree in patients with suspected genetic cardiomyopathy using genetic counseling. In addition, first-degree relatives who are at risk should undergo clinical screening for cardiomyopathy.

## Cardiac Magnetic Resonance

Since the first clinical use of magnetic resonance imaging, there have been major advances in CMR technology that have improved acquisition times, spatial resolution, and temporal footprint. CMR is well known for quantitative evaluation of cardiac structures such as wall thickness, chamber sizes, myocardial mass, and cardiac function. With inline or post-processing software, CMR measurements can be made with high reproducibility and accuracy. In addition, reference values for CMR have been validated and established in the literature [2, 6]. Therefore, CMR can allow for early detection of subclinical cardiac abnormalities before the development of symptoms and close monitoring of cardiac function during treatment periods [7, 8].

CMR can determine myocardial tissue characteristics based on the relaxation properties of protons after a radiofrequency pulse. The relaxation can be measured in the longitudinal (T1) and transverse directions (T2) relative to the magnetic field to the bore of the scanner. Parametric mapping is one technique in which the T1 and T2 relaxation times of the myocardium can be quantified on a pixel-wise basis. Gadolinium-based contrast agents (GBCA) change these relaxation properties based on their accumulation in extracellular space [9].

Native T1 maps are acquired before GBCA administration and relaxation times are commonly elevated in conditions such as acute myocardial injury, necrosis, fibrosis, and infiltrative diseases such as cardiac amyloidosis. Native T1 values can be lower with intramyocardial hemorrhage, thrombus, and fatty infiltration. In post-contrast T1 maps, GBCA shortens the relaxation times in areas of extracellular expansion from infarction, fibrosis, or inflammation. The actual normal values for T1 depend on the local reference ranges of the specific scanner used. The native and post-contrast T1 maps can be combined to estimate the extracellular volume (ECV) of the myocardium, which is an accurate measure of extracellular expansion from interstitial fibrosis. The ECV is a normalized value calculated using myocardial and blood T1 values before and after contrast administration, along with the patient’s hematocrit. Therefore, ECV is less dependent on contrast agent dosing, the timing of measurement post-injection, or renal clearance [9, 10]. In addition, ECV has been shown to correlate better with histological assessments of myocardial fibrosis across different imaging techniques, field strengths, and vendors [8, 11].

T2 mapping measures transverse relaxation time, reflecting the decay of transverse magnetization caused by spin–spin interactions of protons. T2 increases with water content, making it a marker for tissue edema and inflammation. T2* mapping, a related technique, accounts for additional dephasing due to magnetic field inhomogeneities, which is useful for detecting iron overload. Normal values depend on the local reference ranges of the specific scanner used [11].

Late gadolinium enhancement (LGE) imaging is a technique used to visualize focal areas of scars. The pattern, distribution, location, and extent of LGE provide valuable diagnostic and prognostic insights for cardiomyopathy. This involves the administration of GBCA, followed by T1-weighted imaging using an inversion recovery technique. An inversion time (TI) is selected to null the healthy myocardium and optimize the contrast of fibrotic scar. Images are acquired at least 10 min after GBCA is injected to allow adequate time for contrast to wash out from healthy tissue. However, areas of expanded interstitial space will retain the GBCA and appear bright on LGE imaging [10]. Characterization of scars with LGE is one of the most powerful diagnostic and prognostic tools unique to CMR [12, 13].

Stress perfusion imaging with CMR can also be used to rule out ischemic cardiomyopathy and detect microvascular dysfunction in non-ischemic cardiomyopathy. CMR uses first-pass gadolinium images during rest and stress states to detect a reduction in perfusion. Stress is induced pharmacologically with vasodilators such as adenosine, regadenoson, or dipyridamole. In addition to visual assessment, myocardial blood flow (MBF) can be quantified on a pixel-wise basis using dynamic contrast-enhanced imaging combined with tracer-kinetic modeling or deconvolution [14]. These techniques are only reliable when combined with one of the advanced techniques like dual-bolus and dual-sequence imaging and provide precise segmental myocardial blood flow (MBF) [15]. Coronary vasodilatory capacity can also be calculated as the ratio of stress to rest MBF, also known as myocardial perfusion reserve (MPR). In healthy individuals, a normal MPR is greater than 2.5 [14].

## Types of Genetic Cardiomyopathies

### Dilated Cardiomyopathy

Dilated cardiomyopathy (DCM) is a condition in which adverse eccentric remodeling due to non-ischemic disorders results in impaired contractile function. There are currently 19 genes associated with DCM [16, 17]. The location of these gene products is diverse and includes the sarcomere, junctional membrane, Z disc, sarcoplasmic reticulum, desmosome, cytoskeleton, nuclear envelope, ion channel, and RNA binding [3, 17]. A four-year multicenter study found that 32% of genotype-positive DCM patients experienced major events, 16% developed end-stage heart failure, and 20% had malignant arrhythmias [18].

CMR can provide insights into cardiac function, morphology, and tissue characteristics to diagnose and monitor dilated cardiomyopathy. Elevated end-diastolic diameter > 117% of the predicted value adjusted for age and body surface area or dilated ventricular volumes are hallmarks of DCM. CMR can detect subclinical myocardial dysfunction through global ventricular and atrial strain, which is often abnormal in DCM. Myocardial crypts, papillary muscle anomalies, and muscular bands may indicate genetic variants, regional wall motion abnormalities due to fibrosis, and excessive trabeculations secondary to remodeling can be seen in DCM, particularly those involving sarcomeric mutations [19].

In the early stages of DCM, interstitial fibrosis can develop from adverse remodeling, which can be detected based on the elevation of native T1 and ECV. The stepwise increases in T1 values correlate with a higher risk of adverse outcomes [20]. The progression of fibrotic changes can manifest in DCM as linear mid-wall septal LGE, which is associated with an increased risk of all-cause mortality, sudden cardiac death, cardiovascular hospitalization, and ventricular tachycardia in DCM patients [21]. In addition, patients can have a ring-like pattern or transmural involvement of LGE, often linked to DSP, FLNC, and PLN mutations, and have a worse prognosis with a higher risk of arrhythmia [22].

In a meta-analysis of 19 studies (7,330 DCM patients), the authors found LGE to be a better outcome predictor than ejection fraction alone. A 1% increase in LGE extent was associated with a 10% increase in all-cause mortality. LGE location (mid-wall with/without free wall) and pattern (mid-wall, sub-epicardial, and focal) were linked to worse outcomes. The presence of LGE was associated with a higher arrhythmia risk in patients with LVEF ≥ 35% (HR: 5.79) compared to those with LVEF < 35% (HR: 4.49) [12, 23].

There have been imaging features and specific gene-associated DCM subtypes [19]. One subtype of interest is LMNA cardiomyopathy. CMR identifies interstitial fibrosis as an early marker, preceding clinical symptoms and LGE which are seen in advanced disease. Mid-wall LGE indicates a poor prognosis. Elevated ECV is found in LMNA mutation carriers with normal LV function and in some without LGE. Other gene-specific LGE and CMR findings are shown in Table 1.

**Table 1:** Types of genetic cardiomyopathies with known associated genetic mutations/alleles, cellular locations or associated proteins, patterns of late gadolinium enhancement (LGE), and advanced cardiac magnetic resonance (CMR) features, including parametric mapping (T1, T2, T2* and extracellular volume (ECV) fraction values)

Genetic Cardiomyopathies: Cellular Locations or Proteins or Phenotypes	Associated Genes	Disease/Gene Specific CMR LGE Findings	Other CMR Specific Features or Parametric Mapping
Dilated Cardiomyopathy (Cellular locations/Proteins)		Mid myocardial LGE	LVEDV > 117% predicted, Decreased LVEF
Sarcomere	*TTN, TTNtv, TNNT2, ACTC1, MYBPC3, MYH6, MYH7, ACTC1, FHL1, FHL2, ANKRD1, TNNC1*	*TTN* -mid wall septum and lateral segments	Myocardial crypts, papillary muscle anomalies, muscular bands and excess trabeculation
Z—disc	*FLNC, DES, DMD, NEXIN LDB3, MYPN, CSRP3, NEBL, VCL, TCAP*	*FLNC-*subepicardial inferolateral wall	Native T1 elevated (stepwise elevated worse prognosis) *LMNA*—elevated ECV T2 elevated with inflammatory/myocarditis presentation
Desmosome	*DSP, DSC2, DSG2, PKP2, JUP*	Mid/subepicardial lateral/inferior lateral. *DSP/PKP2* hot phase as ring like midmyocardial can involve RV wall	
Laminin A/C	*LMNA*	Basal septum mid wall	
Ion Chanel	*SCN5 A, RYR2*	Limited LGE	
Cytosol	*EEF1 A2, BAG3 (Z-line)*	Diffuse mid wall (basal & mid segments)	
Nucleus	*PRDM 16, TBX 20, RBM 20*	Patchy mid myocardial septal > lateral wall	
Hypertrophic Cardiomyopathy (Cellular locations/Proteins)			LV thickness > 13 − 15 mm Phenotypes- Isolated basal septum, reverse curvature, apical, mid cavity obstruction, rare- concentric, RV predominant and lateral wall
Thick Myofilament protein	*MYH7, MYBPC3, MYH7B, MYL2, MYL3, TTN, MYH6*	Mid wall, *MYH7*- septum & LV free wall. *TTN* – subepicardial or mid wall *MYL*- apical	Preclinical—elongation of the anterior leaflet, myocardial clefts, and abnormal papillary muscles Elevated T1 and ECV in the area of hypertrophy Aneurysm, thrombi Low MBF and MPR like in CMD *MYH7, MYBPC3*—Reverse Curvature hypertrophy (HCM) *MYBPC3, ACTC1* – Apical HCM *MYH7, MYBPC3, TNNI3*—Mid-Cavity Obstruction *ALPK3*—early dilated later apical concentric HCM *PRKAG2* – inferolateral, septum hypertrophy and abnormal glycogen storage, T1 low early and high later, T2 normal
Thin Myofilament protein	*TNNT2, TNNI3, TNNI3 K, TPM1, ACTC1*	*ACTC1*-subepicardial apical *TNNT2, TNNI3* – more diffuse	
Z-discs	*MYOZ2, ACTN2, CSRP3, TCAP, FHL 1, MYPN, NEXN*	*ACTN2, CSRP3-*anteroseptal, inferolateral. *MYOZ2* – septum, LV free wall. *TCAP* – apical	
M band	*OBSCN, TRIM63, MYOM2*	limited info *OBSCN* subepicardial	
Additional sarcomere	*FLNC, ALPK3, CAV3, CRYAB*	Patchy mid wall to extensive extend to lateral wall	
Non sarcomere	*PRKAG2, CAV1, KCNQ1*	Patchy diffuse mid-inferolateral, septum	
Calcium Homeostasis	*JPH2, PLN*	*PLN*-inferolateral	
Nucleus	*LMNA, GATA4, NKX2 - 5*	Sub-epi/endocardial in the hypertrophy area	
Arrhythmogenic Cardiomyopathy (Phenotypes)			Regional RV akinesia or dyskinesia or dyssynchronous RV contraction RV EDV/BSA ≥ 110- 100 mL/m2 (male) or ≥ 100 − 90 mL/m2 (female) RVEF ≤ 40%− 45%
RV predominance	*PKP2, TGFB3, CTNNA 3, CDH2, THEM43, RYR2, JUP*	Subepicardial/transmural RV free wall *TMEM43*-LV subepicardial	RV/LV dilation or decreased function, dyskinesis Variable T1 values (elevated in fibrotic and decreased in fat infiltration areas) *LMNA* – elevated ECV Low T1 with fat infiltration area and elevated T1after fibrotic changes T2 elevated with active inflammation, decreased in areas of fat infiltration
LV predominance	*DSP, DMD, Duchenne, DMD Becker, DMPK*	*DSP-* LV lateral wall, DMD- mid wall inferolateral, *DMPK*—septal/lateral wall	
Biventricular	*DSP, DSC2, DSG2, PLN, FLNC, DES, LMNA, PKP3, JUP, CAV3*	*DSP* mid wall ringlike, *DSC2* subepicardial LV, diffuse RV *PLN*—diffuse mid wall LV *FLNC* patchy spares apex,	
Non-Dilated Left Ventricular Cardiomyopathy (NDLVC)	*DSP, FLNC-truncating variants, DES, LMNA, PLN, THEM43, RBM20*	Mid-wall or subepicardial inferior lateral wall, rarely circumferential “ring like”	Non-dilated ventricles, hypokinesis, fatty infiltration, elevated T2 with inflammation and edema
Left Ventricle Hypertrabeculation Morphologic trait	*MYH7, MYBPC3, TAZ, DSP, LDB3, SCN5 A, ACTC1, TMNT2, CAV3, NEXN, VCL, PRKAG2, LMNA*	Trabeculated area common site inferolateral LV wall	Increased trabeculation with non-compacted to compacted myocardial ratio ≥2.3, elevated T1 and ECV in area of fibrosis
Infiltrative Cardiomyopathy			
Iron overload Cardiomyopathy	*HFE1,2,3, HJV, HAMP, TFR1,2, SLC40 A1, FTH1 hemoglobinopathies*	Midmyocardial and subepicardial LV free wall/septum Usually, apex spared and rarely RV involved	Dilated/Restrictive Phenotype, T2* decreased < 10 diagnostic for iron overload Low T1 and T2 values, normal ECV
Inherited Cardiac Amyloidosis	*TTR gene variants*	Diffuse subendocardial or transmural	Hypertrophy with infiltration, significantly elevated T1 and ECV values Lower T2 to skeletal muscle ration, abnormal circumferential stain
Fabry Disease	*GLA*	Mid myocardial inferolateral and later other areas or RV	Increased LV, RV mass, later decreased function, Low T1 & elevated T2 that reverses with enzyme replacement
Peripartum cardiomyopathy	*TTN, BAG3, DSP, FLNC*	Patchy sub-epicardial, mid myocardial inferior, lateral, to mid-anteroseptal LV walls	LVEF < 45%, elevated T2 values
Muscular dystrophy related cardiomyopathy	*DMD, SGCA, EMD, ACADVL, LMNA, TAZ, LAMP2, TCAP, CRYAB*	Subepicardial inferolateral wall	DMD, BMD, DM—Elevated T1, T2 & ECV values

LVEF – left ventricle ejection fraction, RVEF- Right ventricle ejection fraction, LV—Left ventricle, RV—Right ventricle, LVEDV-left ventricle end-diastolic volume, RVEDV- Right ventricle end-diastolic volume, BSA- Basal surface area, MBF – Myocardial blood flow, MPR – Myocardial perfusion reserve, CMD – Coronary microvascular dysfunction

Genes glossary: TTN—Titin, TTNtv – Titin truncating variants, TNNT2—Troponin T2, Cardiac Type, TMNT2—Actin Alpha Cardiac Muscle, MYBPC3—Myosin Binding Protein C3, MYH6—Myosin Heavy Chain 6, MYH7—Myosin Heavy Chain 7, FHL1—Four and a Half LIM Domains 1, FHL2—Four and a Half LIM Domains 2, ANKRD1—Ankyrin Repeat Domain 1, TNNC1-Cardiac troponin C, FLNC—Filamin C, DES—Desmin, DMD—Dystrophin, NEXIN—Nexilin, LDB3—LIM Domain Binding 3, MYPN—Myopalladin, CSRP3—Cysteine and Serine-Rich Protein 3, NEBL—Nebulin-like Protein, VCL-Vinculin, TCAP-Telethonin, MYPN-Myopalladin, KCNQ1-Potassium voltage-gated channel subfamily member1, GATA4-GATA binding protein 4, NKX2 - 5- NK2 homeobox5, THEM43-Transmembrane protein 43, RYR2-Ryanodine receptor2, JUP-Junction plakoglobin, DSP—Desmoplakin, DSC2—Desmocollin 2, DSG2—Desmoglein 2, PKP2—Plakophilin 2, JUP—Junction Plakoglobin, OBSCN—Obscurin, TRIM63—Tripartite Motif Containing 63, MYOM2—Myomesin 2, JPH2—Junctophilin 2, PLN—Phospholamban, ALPK3—Alpha-Kinase 3, PRKAG2—**Protein Kinase AMP-Activated Non-Catalytic Subunit Gamma 2**, CAV3—Caveolin 3, CRYAB—Crystallin Alpha B, DMPK—Dystrophia Myotonica Protein Kinase, SGCA—Sarcoglycan Alpha, EMD—Emerin, ACADVL—Acyl-CoA Dehydrogenase, Very Long Chain, LMNA—Lamin A/C, PRDM 16-Positive regulatory domain zinc finger region protein 16, TBX 20 – T-box trascripyion factor 20, RBM20-RNA binding motif protein20, HFE- Human hemochromatosis protein gene, HJV- Juvenile hemochromatosis gene, HAMP- Hepcidin antimicrobial peptide gene, TFR2- Transferrin receptor 2 gene, SLC40 A1- Solute carrier family 40 member 1, FTH1-Ferritin heavy chain1, TTR- Transthyretin, GLA- Galactosidase Alpha, SCN5 A- Sodium Voltage-Gated Channel Alpha Subunit 5 A, NEXN-Nexillin F-actin binding protein, BAG3-Bcl- 2-Associated Athanogene 3 TAZ—Tafazzin, LAMP2—Lysosomal-Associated Membrane Protein 2

DCM can resemble myocarditis with similar LGE patterns and elevations in T2. Pathogenic genetic variations can be seen in about 22% of adults and 44% of children with myocarditis and concomitant heart failure or ventricular arrhythmias [24]. This observation is thought to arise from the interaction between genetic predispositions and environmental triggers like infections [25]. For example, TTN truncating variants may impair the stress response, contributing to the transition from inflammation to full-blown DCM, while mutations in the DSP gene, common in arrhythmogenic cardiomyopathy, may predispose the heart to inflammation due to defects in cardiac desmosomes. In these cases, myocardial inflammation could act as a"second hit,"triggering or exacerbating the phenotypic expression of genetic cardiomyopathies [24]. These CMR and genetic findings suggest a possible inflammatory pathway leading to DCM and a potential therapeutic target that requires further exploration.

### Hypertrophic Cardiomyopathy

Hypertrophic cardiomyopathy (HCM) is the most common inherited cardiomyopathy with a prevalence of 1:200–1:500. HCM is characterized by maximal left ventricular (LV) wall thickness ≥ 15 mm without another cause, or ≥ 13 mm with a family history or genotype. Thought of as a genetic disease of the sarcomere, over 1400 mutations have been identified in at least 8 causative genes. The most common genes are myosin heavy chain 7 (MHY7) and myosin-binding protein C (MYBC3). Despite this, only ~ 40% of patients have an identifiable genetic mutation. Another 20–30% exhibit autosomal dominant inheritance without a clear causative gene [26].

**Fig. 1:**
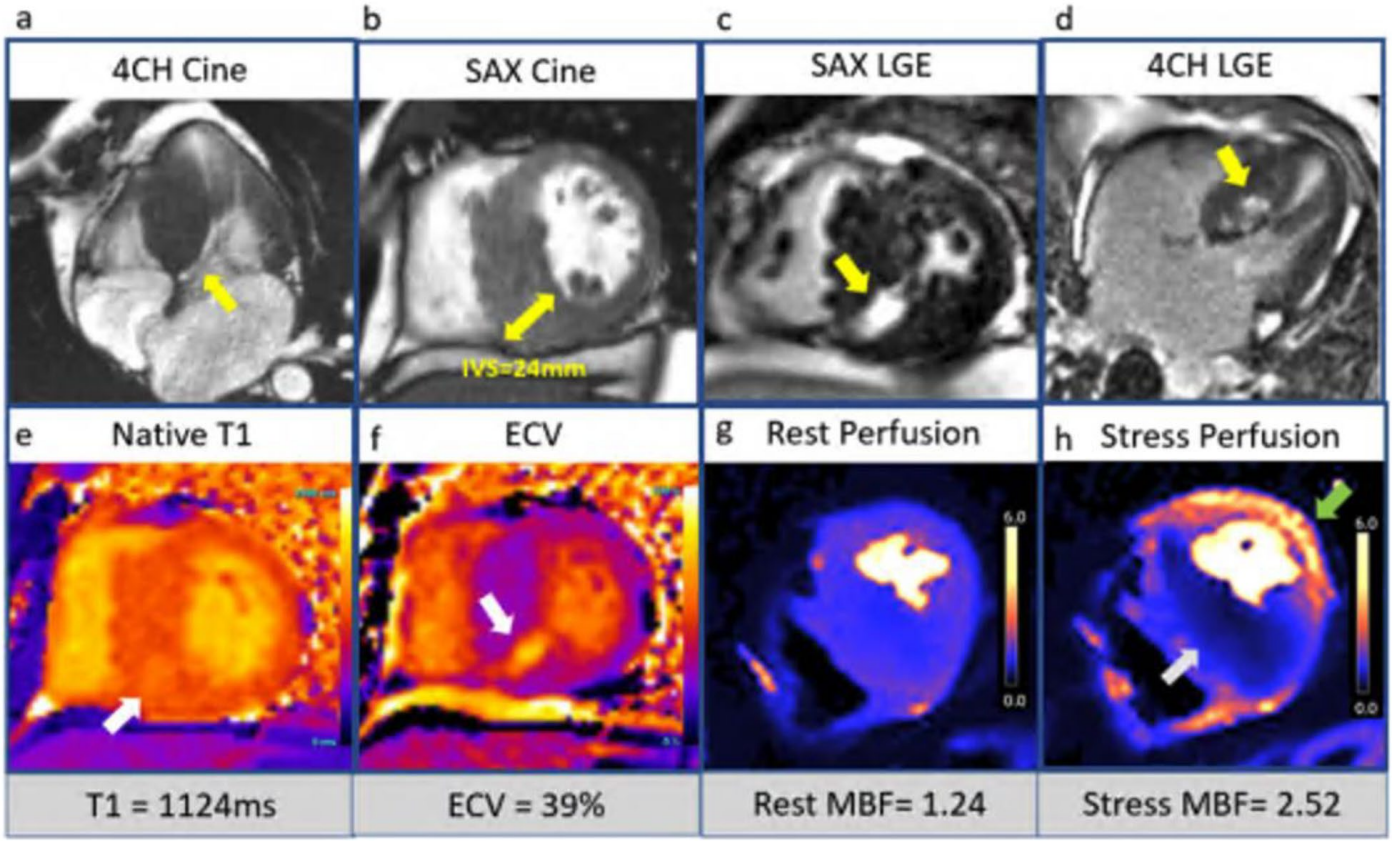
Hypertrophic cardiomyopathy. Reverse curvature phenotype hypertrophic cardiomyopathy stress CMR: **a)** Systolic anterior motion of the anterior mitral valve leaflet (SAM) observed on SSFP cine image on 4 CH (4 Chamber) view (*yellow arrow*). **b)** maximum wall thickness of 24 mm in the mid-short axis (SAX) cine image in the end of diastole (*yellow arrow*). **c)** short axis phase-sensitive inversion recovery (PSIR) late gadolinium enhancement (LGE) image: dense patchy LGE observed in the mid inferoseptum (*yellow arrow*). **d)** long axis PSIR LGE image: patchy LGE in the mid inferoseptum on 4 CH view (*yellow arrow*). **e)** native T1 mapping showed increased T1 relaxation time (1124 ms) in the mid inferoseptum (*white arrow*). **f)** Extracellular volume (ECV) image showed increased ECV value (39%) in mid inferoseptum (*white arrow*). **g)** rest perfusion pixel-wise mapping image with global resting myocardial blood flow (MBF) of 1.24 ml/g/min. **h)** stress MBF pixel-wise mapping image with low stress MBF (0.81 ml/g/min) in the mid septum (*white arrow*) and normal stress MBF (2.52 ml/g/min) in the mid lateral walls (*green arrow*), Myocardial perfusion reserve (MPR) in mid septum: 0.85, MPR in mid lateral walls: 2.31

According to the Hypertrophic Cardiomyopathy Registry, there are two main subtypes of HCM. The first is the isolated basal septal hypertrophy subtype, which accounts for 40–50% of cases. In this group, pathogenic mutations are rare (~ 10%) and patients are typically older. In addition, they have more left ventricular outflow tract obstruction but lower sudden cardiac death (SCD) risk. The second is the reverse curvature hypertrophy subtype. This group is characterized by mid-septal thickening and a counter-clockwise “spiraling” hypertrophy pattern as the myocytes traverse from the LV base to the apex as seen in the short axis plane of the ventricle. Hypertrophy starts at the basal anterior wall, increasing to maximal thickness in the mid-septum, and then continuing into the mid-inferior segments (Fig. 1). Up to 90% of this subtype has an identifiable genetic mutation. There is a lower incidence of left ventricular outflow tract obstruction but a higher SCD risk [27]. A rarer subtype is apical HCM, which accounts for 5–10% of cases in the United States. They can have a favorable prognosis, but the development of mid-cavity obstruction and aneurysm drastically increases the risk of thrombus and arrhythmias [28, 29]. Additional HCM subtypes include those with concentric hypertrophy, neutral septal pattern, right ventricle (RV) predominance, and isolated lateral wall involvement [29].

According to the American College of Cardiology (ACC), American Heart Association (AHA), and European Society of Cardiology (ESC) 2023 guidelines, CMR has a class I recommendation for evaluating HCM. Specifically, CMR can be used to confirm myocardial thickness, quantify LGE burden, assess ejection fraction, help with procedural planning, and rule out alternative diagnoses [17, 30]. CMR combined with genetic testing can differentiate HCM from mimics like hypertensive or athletic remodeling, amyloidosis, and other cardiomyopathies. For patients with borderline maximal LV thickness that do not meet the criteria of HCM, additional CMR features may provide supporting evidence for a sarcomere disorder. Findings such as elongation of the anterior leaflet, myocardial clefts, and abnormal papillary muscles may be antecedent features in HCM [31]. Regarding specific genes, ALKP3 truncating variants can often be identified based on their distinct phenotypes. Heterozygotes show mixed concentric and apical hypertrophy with extensive LGE, while homozygotes develop early-onset dilated cardiomyopathy and later HCM-like hypertrophy [32]. However, CMR cannot distinguish between the more common genotypes such as MYH7 and MYBPC3.

Data on genotype and outcomes are mixed, but CMR LGE imaging of scars is an important prognostication tool. Most agree that sarcomere mutation-positive patients exhibit more fibrosis on CMR [33]. Ninety percent of HCM cases involve the septum, especially the commonly thickened segments such as the basal anterior or anteroseptal regions (Fig. 1). When quantifying replacement fibrosis in HCM with CMR [25, 26], LGE greater than 10–15% of the myocardial mass is associated with a higher mortality risk. CMR can also detect ventricular aneurysms and assess systolic function, which can help guide decisions regarding implantable cardioverter-defibrillator (ICD) placement according to ACC/AHA guidelines [30]. Deep learning has also been applied to CMR and HCM to identify scars without the need for GBCAs, which is known as virtual native enhancement imaging. This technique could help reduce cost and scan time while improving accessibility for HCM patients [34]. Quantitative CMR perfusion imaging has also been studied in HCM, demonstrating microvascular dysfunction based on reduced MBF and MPR (Fig. 1). Interestingly, these abnormalities are observed even in regions without hypertrophy or fibrosis [35].

### Arrhythmogenic Cardiomyopathy

Arrhythmogenic cardiomyopathy (ACM) is a genetic myocardial disease characterized by fibro-fatty replacement of myocardium and associated ventricular arrhythmias and sudden cardiac death. CMR is the imaging modality of choice due to its ability to provide detailed morpho-functional and tissue characterization of both ventricles. There are multiple criteria for diagnosing ACM. The 2010 Task Force criteria are specific for AMC with primarily RV involvement, while the 2020 Padua criteria also include left-dominant phenotypes with additional CMR structural abnormalities [36, 37]. The European Task Force consensus report published in 2024 proposed incorporating more LGE-based CMR findings, increased recognition of left-sided and biventricular phenotypes, and routine integration of genetic findings [22, 36].

**Fig. 2:**
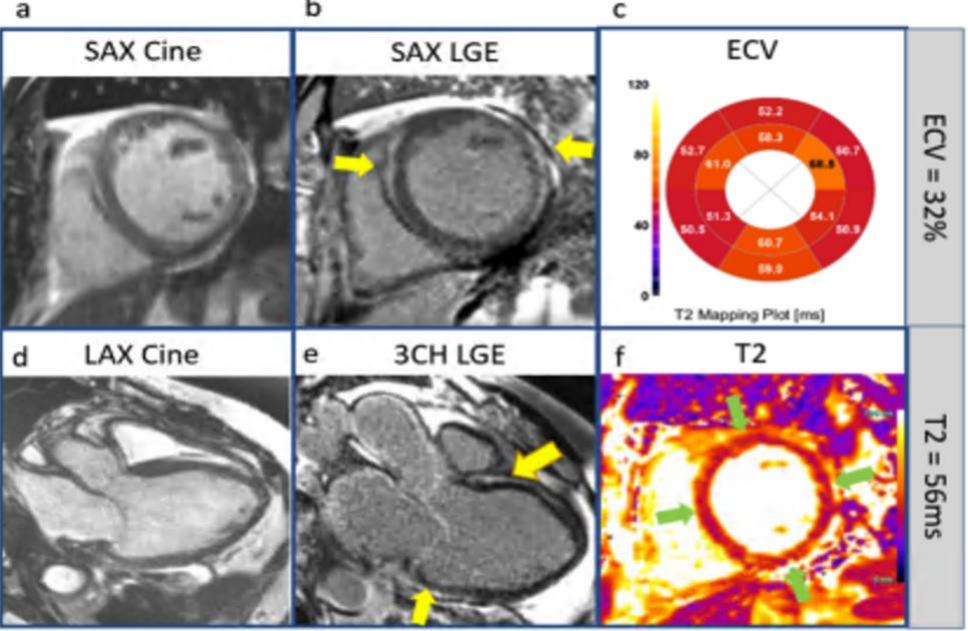
Arrhythmogenic cardiomyopathy with DSP pathogenic gene: **a)** short axis (SAX) cine image, **b)** short axis late gadolinium enhancement (LGE) image: LGE stripe in the mid septum (*yellow arrow*). **c)** ECV (extracellular volume) mapping showed diffusely increased ECV (32%), **d)** long axis (LAX) 3 chamber (3 CH) cine showed severely enlarged left ventricular size and LVEF 26%. **e)** long axis phase-sensitive inversion recovery (PSIR) LGE image: mid wall stripe of LGE in septum on 3 CH view (*yellow arrows*). **f)** T2 image showed increased T2 value (56 ms) (*green arrows*)

There are many CMR features to evaluate for individuals with suspected ACM. RV-predominant phenotypes can have LGE primarily in basal RV regions (lateral/inferior walls), sparing the apex. In the RV, wall motion abnormalities like akinesia, dyskinesia, or aneurysms may occur. However, the relatively thin wall of the RV compared to the LV can decrease the sensitivity of these findings. Therefore, combining LGE information with motion abnormalities is recommended to improve diagnostic accuracy. In LV-predominant ACM, LGE typically appears as subepicardial or mid-myocardial scarring (Fig. 2), especially in the inferolateral wall. Both LV and RV ACM can have regional hypokinesis despite normal ventricular volumes. Biventricular ACM combines both features RV and LV phenotypes, with LGE and segmental wall motion abnormalities extending to both chambers. Aquaro et al. demonstrated CMR detected ACM with biventricular involvement is associated with a worse prognosis, with an increased risk of fatal arrhythmias compared to RV involvement alone. However, patients diagnosed with ACM based on the revised Task Force criteria but no evidence of the disease on CMR had a favorable prognosis with no major events over 5-years [40].

The different subtypes of ACM are associated with distinct genetic variants. RV involvement is seen in genetic mutations such as Plakophilin- 2 (PKP2), Desmocollin- 2 (DSC2), and Junction Plakoglobin (JUP). LV predominance is associated with Desmoplakin (DSP) (Fig. 2), Lamin (LMNA), Filamin C (FLNC), and Transmembrane Protein 43 (TMEM43). Finally, biventricular involvement occurs with genes such as Desmoglein- 2 (DSG2), Desmin (DES), and Phospholamban (PLN) [38]. Variants in the *TMEM43* gene were associated with a high prevalence of subepicardial late gadolinium enhancement and left ventricular dysfunction on CMR images [39].

Research has shed light on the role of inflammation and immunopathology in ACM and other genetic cardiomyopathies. Histological studies, reported by Thiene and Basso, reveal inflammatory infiltrates of lymphocytes surrounding necrotic myocytes in two-thirds of ACM hearts, indicating immune activation. It is postulated that autoimmune mechanisms may further amplify disease progression. Validated across studies, increased DSG2 autoantibodies have been shown to result in impaired gap junction function and increased arrhythmia burden [40]. In ACM, TGFβ3 signaling is an important driver of fibrotic remodeling, while NFκB and GSK3β pathways exacerbate inflammatory responses and desmosomal instability [41]. A multicenter study by Corrado et al. found that PKP2 dysregulation is a key link between cytokine release and structural destabilization [42]. A study by Peretto et al. showed that immunomodulatory therapy can potentially be used to treat inflammation in cardiomyopathies linked to the DSP (Fig. 2), TTN, FLNC, PKP2, LMNA, and SCN5 A genes. Based on imaging and biopsy, they demonstrated that immunomodulatory therapy cleared inflammation in 67% of subjects, potentially reversing genetic inflammatory pathways [43].

### Inherited Cardiac Amyloidosis

Hereditary transthyretin amyloidosis (ATTR) is caused by an autosomal-dominant transthyretin gene variant, which leads to progressive misfolded protein fibrils extracellular deposit. When there is cardiac involvement, ATTR can cause thickening of the myocardium, impaired diastolic function, and restrictive physiology, which results in heart failure and adverse cardiovascular events. For hereditary ATTR, 2023 ACC expert consensus recommends regular monitoring in asymptomatic gene carriers, starting about 10 years before the proband's onset age followed by testing every 3–5 years unless symptoms appear [44–46]. CMR is one of the modalities recommended for the evaluation of suspected cardiac amyloidosis.

**Fig. 3:**
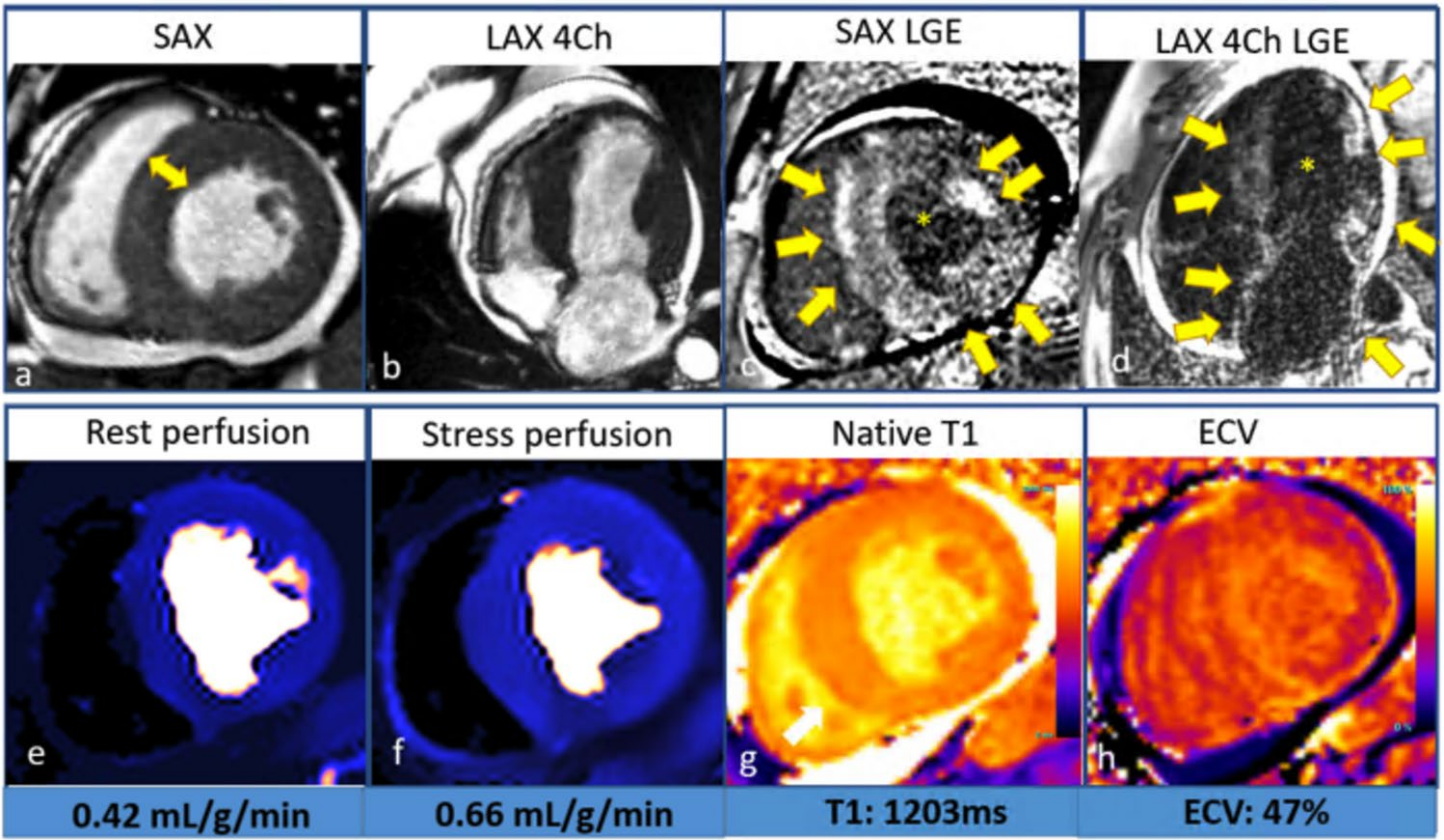
Inherited cardiac amyloidosis. Transthyretin amyloid (ATTR) stress cardiac magnetic resonance imaging showed **a)** moderate left ventricle (LV) concentric hypertrophy (*arrow* illustrates interventricular septum = 17 mm measured at the short axis (SAX) basal slice at diastole), **b)** severely reduced LV ejection fraction (LVEF 17%) as illustrated on the long-axis (LAX) four-chamber (4 CH) view at systole; Diffuse late gadolinium enhancement (LGE) in nonischemic pattern as indicated by *multiple arrows* **c)** in short axis phase-sensitive inversion recovery (PSIR) LGE image and **d)** in the four-chamber magnetization-prepared inversion recovery (MagIR), with the latter also showing left atrial LGE. In both views, the nulled blood pool signal can be appreciated as indicated by an *asterisk* (*); **e)** abnormal myocardial blood flow (MBF) at rest (0.42 mL/g/min);** f)** markedly global reduced stress MBF (0.66 mL/g/min) and myocardial perfusion reserve (MPR 1.57). **g)** Native T1 mapping showed globally increased T1 relaxation time of 1203 ± 5.05 ms (1.5 T) and **h)** elevated extracellular volume (ECV) fraction (47%)

CMR is a robust imaging tool to identify the presence, location, and distribution of hypertrophy and detect cardiac amyloid infiltration. A TI-scout (inversion time) determines the optimal nulling time of normal myocardium after gadolinium contrast. Amyloid deposits expand the extracellular space, causing faster gadolinium washout from the blood pool than the myocardium. This reverse nulling pattern has a specificity for cardiac amyloidosis. The pattern of LGE is classically subendocardial or transmural, with very elevated ECV and T1 mapping values providing high sensitivity for amyloid infiltration (Fig. 3). ATTR cardiomyopathy will frequently present with increased LV mass and either low normal or reduced LVEF. The LGE pattern can involve RV and atria. In genetically positive patients, a reduction in circumferential strain can be seen in early amyloid infiltration with high sensitivity.

Elevated ECV and native T1 times are associated with higher amyloid burden, interstitial expansion, and risk of death. In a meta-analysis by Cai et al., each 3% increase in myocardial extracellular volume (ECV) on CMR was associated with a 16% higher mortality risk, and every 60 ms rise in myocardial T1 time corresponds to a 33% increased mortality risk [47].

CMR quantitative perfusion is also abnormal in cardiac amyloidosis. Patients have severely reduced stress MBF and MPR (Fig. 3), correlating with amyloid infiltration, myocardial changes, and disease severity. Therefore, the evaluation of microvascular dysfunction with quantitative CMR perfusion may be valuable for diagnosis, prognosis, and monitoring treatment response [48].

### Iron Overload Cardiomyopathy

Iron overload cardiomyopathy can result from primary genetic disorders like hereditary hemochromatosis (four types: HFE, HJV, HAMP, TfR2, and SLC40 A1 mutations), which lead to excessive iron absorption and cardiac accumulation. Secondary causes include autosomal recessive hemoglobinopathies such as thalassemia major (with over 350 mutations on chromosome 11) and sickle cell anemia (which is characterized by a point mutation where valine replaces glutamic acid) as a less common cause. These conditions require repeated blood transfusions, causing iron buildup in the heart. Iron overload induces oxidative stress, mitochondrial dysfunction, and impaired calcium handling, leading to myocardial damage [49].

**Fig. 4:**
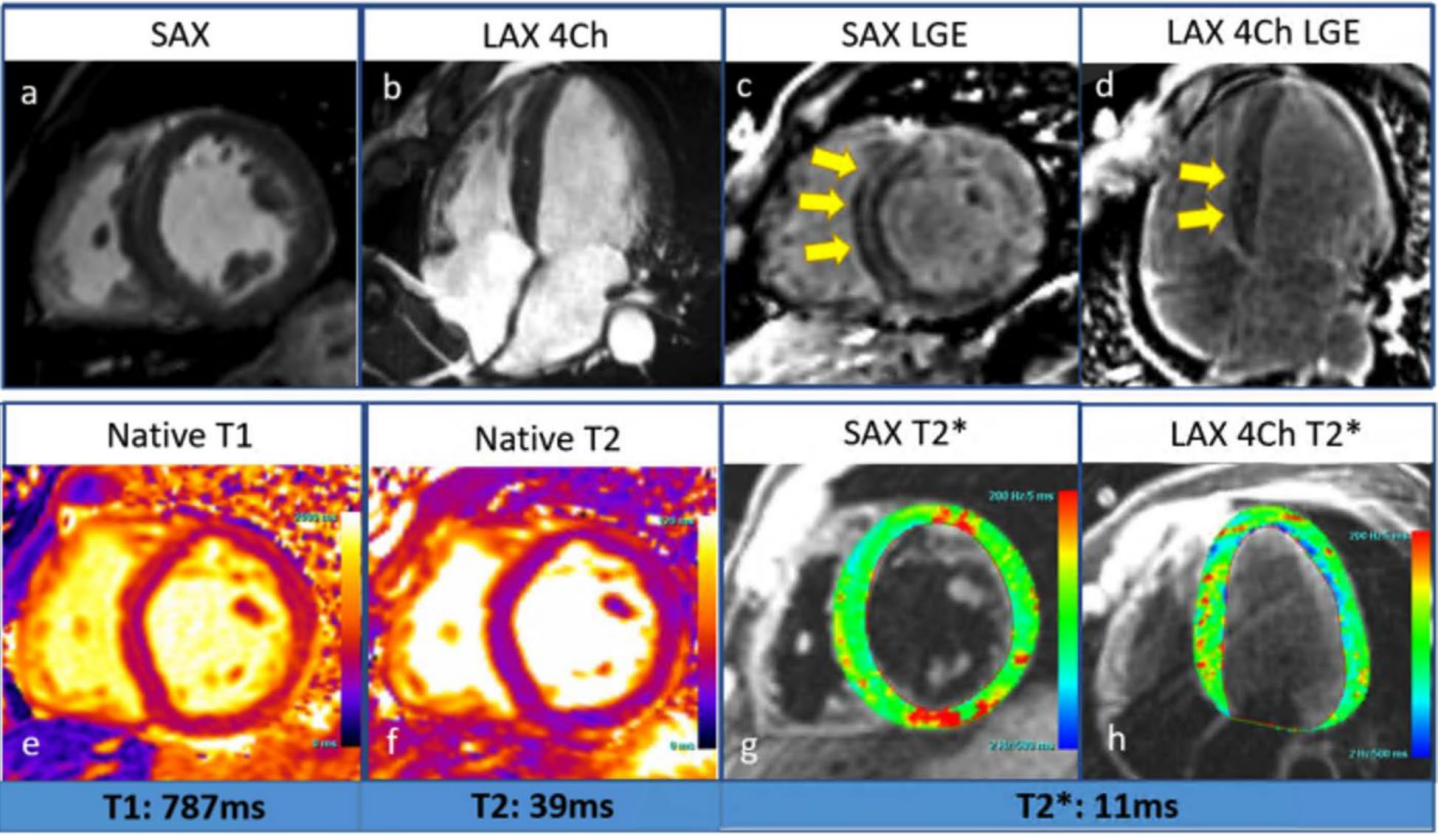
Iron overload cardiomyopathy **a)** Moderately dilated size (end-diastolic volume indexed: 123 ml/m^2^) and normal wall thickness interventricular septum = 8.5 mm measured at the short axis (SAX) basal slice at diastole) and severely reduced left ventricle systolic function (LVEF: 28%), **b)** four-chamber (4 Ch) long-axis (LAX) view at systole. There was evidence of mid-wall stripe of late gadolinium enhancement (LGE) in the basal septum and focal epicardial LGE in the basal inferior segment consistent with myocardial fibrosis as illustrated by *arrows* on the **c)** short axis and **d)** four-chamber magnetization-prepared inversion recovery (MgIR) image. **e)** Native mapping showed globally reduced T1 relaxation time, elevated ECV (42%), and **f)** reduced T2 relaxation time. **g and h)** Myocardial T2* map quantification (R2* 94.4 Hz) indicated cardiac iron overload status

CMR is the gold standard for detecting myocardial iron accumulation and associated dysfunction. T2* relaxometry measures transverse relaxation time; lower values (< 20 ms) indicate iron overload, with values < 10 ms in severe cases associated with an increased risk of ventricular arrhythmias and heart failure Fig. 4. However, T2* can be affected by motion artifacts and may be less reliable in mild iron overload. T1 mapping offers a more sensitive alternative, measuring longitudinal relaxation time. CMR aids in guiding iron chelation therapy, with T2* values informing treatment response and intensity, while also monitoring disease progression, correlating with LVEF, and detecting early diastolic dysfunction in primary and transfusion-dependent iron overload patients [50, 51, 52].

### Anderson-Fabry Cardiomyopathy

Anderson-Fabry disease (AFD) is a rare X-linked multisystem genetic disorder with α-galactosidase deficiency leading to abnormal accumulation of glycosphingolipids within lysosomes [53]. Early detection is essential for optimal treatment and to prevent fibrosis in the later stages of the disease. Key CMR features include myocardial hypertrophy, lower T1 mapping values with early glycosphingolipid accumulation, and elevated T2 mapping values with inflammation. CMR detects early changes by measuring an LV global longitudinal and atrial strain before LV hypertrophy or lower T1 values occur. LV hypertrophy and LGE seen on CMR is associated with a higher cardiac event, while their absence suggests a better prognosis [54, 55]. CMR can also be used to monitor treatment response to enzyme replacement therapy. In patients with baseline hypertrophy but minimal LGE, enzyme replacement has been shown to decrease LV wall thickness and mass on CMR. In addition, normalization of T1 and T2 relaxation times correlates with decreasing wall thickness and LV mass. However, enzyme treatment has not been shown to change RV mass and LGE burden [56].

#### Non-Dilated Left Ventricular Cardiomyopathy (NDLVC)

Since 2023, the ESC classified a separate and distinct category of non-dilated left ventricular cardiomyopathy (NDLVC) defined as a cardiomyopathy with non-ischemic LV scarring or fatty replacement without dilation. It may be accompanied by wall motion abnormalities/hypokinesis that is independent of hypertension, valvular disease, or coronary artery disease [17]. It encompasses early-stage DCM, non-dilated hypokinetic forms, and arrhythmogenic LV involvement; however, its prevalence and management are still underexplored. NDLVC shares genetic overlap with DCM and ARVC (Table 1). Mutations can affect severity, progression, and SCD risk. Genetic screening is essential for diagnosis, risk stratification, and family evaluation [17]. CMR is important for diagnosing NDLVC and also for risk stratification, detection of non-ischemic fibrosis, scarring, and adipose replacement. Mid-wall distribution of LGE is a major risk marker and the LGE patterns vary by genetics. T2 mapping identifies edema in inflammatory cases [57]. Eda et al. also found NDLVC in 22% of non-ischemic cardiomyopathy, with these individuals having more AF, less LGE (34% vs 53%), and similar outcomes when compared to DCM [58]. Progression to dilated cardiomyopathy predicted worse prognosis, highlighting the need for serial imaging and risk assessment. A recent retrospective study of 23,472 CMR scans found ring-like LGE in 1.1% of cases. It was a high-risk marker for adverse outcomes and arrhythmias, especially when ≥ 7 segments were involved. This pattern was commonly linked to desmosomal (DSP) and non-desmosomal (FLNC, LMNA) genes with both NDLVC and DCM and less often associated with ARVC, HCM, or other cardiomyopathies [59].

### Left Ventricular Hypertrabeculation

**Fig. 5:**
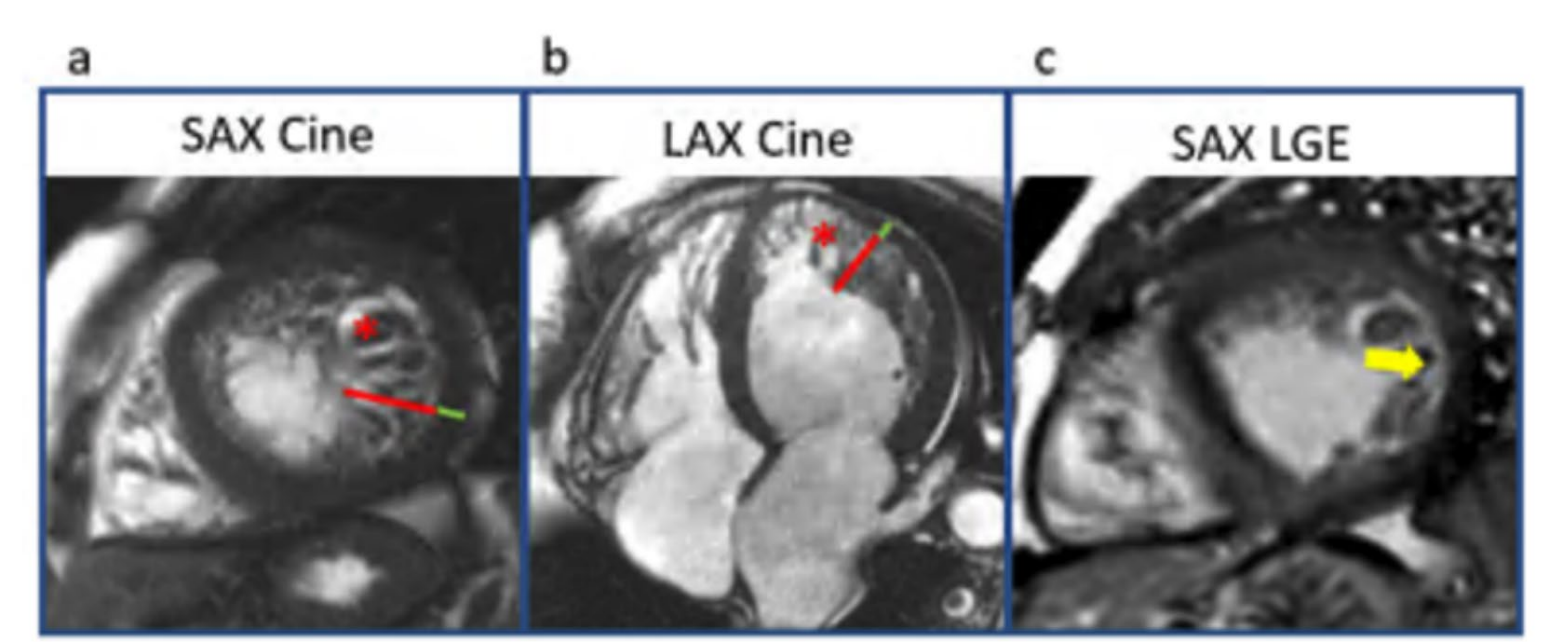
Leftventricular hypertrabeculation in dilated cardiomyopathy. **a)** short axis (SAX) cine image, **b)** 4 chamber long axis (LAX) cine image at the end of diastole shows dilated left ventricle with highly trabeculated myocardium, which is distributed at the apex and mid anterior and lateral walls (*red asterisk*). The ratio of non-compaction (*red line*) and compaction (*green line*) is > 2.3. **c)** short axis phase-sensitive inversion recovery (PSIR) late gadolinium enhancement (LGE) image: subendocardial LGE in the mid-lateral wall (*yellow arrow*)

Left ventricular non-compaction (LVNC), once considered a separate cardiomyopathy, is now recognized as a morphologic trait. Excessive trabeculation can be seen in up to 20% of healthy individuals and in physiologic states such as athletic training or pregnancy [60]. When present with cardiac disease, it often overlaps with other cardiomyopathies, including DCM, NDLVC, ARVC, or other genetic forms (Fig. 5). Over 80 genes have been linked to excessive trabeculation, involving sarcomere structure, mitochondrial function, X-linked disorders, and cardiac development. Hypertrabeculation is also found in 20–30% of patients with Duchenne or Becker muscular dystrophy and is associated with worse outcomes [61]. A non-compacted to compacted (NC/C) myocardial ratio ≥ 2.3 suggests hypertrabeculation is better assessed by CMR and if present the CMR can further, identify underlying phenotypes with specific cardiomyopathy diagnosis. Adverse outcomes are associated with lower LVEF, higher NC/C ratio, enlarged LV volumes, increased mass, fibrosis, and LGE—particularly in genetic variant carriers. CMR-derived atrial strain also helps identify individuals with multiple genetic variants [62].

### Peripartum Cardiomyopathy

Peripartum cardiomyopathy is defined as idiopathic heart failure with a reduced ejection fraction (LVEF < 45%), occurring during the last month of pregnancy or within five months of the postpartum period. Recent studies have shown genetic links in peripartum cardiomyopathy, such as single-nucleotide polymorphisms near the PTHLH gene, along with TTN and BAG3 [63]. There was a 15% prevalence of truncating variants (i.e., nonsense, missense, or splicing variants), with two-thirds of TTN variants associated with a poor prognosis [64]. TTN truncating mutations occurred in 10.4% of PPCM women (vs. 9.4% reference), associated with FLNC, DSP, and BAG3 genes and this group had lower LVEF [65].

CMR is safe during pregnancy, however gadolinium-based contrast agents can only be used after delivery. In clinical practice, CMR with contrast is obtained postpartum and usually later in the disease course which is still crucial for assessing prognosis. A meta-analysis has shown that the presence of LGE, with a prevalence of 67.5%, is a significant prognostic marker for poor LV function recovery, while myocardial edema (elevated T2) with a prevalence of 16.6%, is less predictive and mostly noted in the early stage [66].

### Muscular Dystrophy Related Cardiomyopathy

Muscular dystrophies (MDs) are inherited disorders impacting the structure and function of different muscles. They often affect the heart due to shared proteins between cardiac and skeletal muscles. Common MD cardiomyopathies include Duchenne MD, Becker MD, Emery-Dreifuss MD, myotonic dystrophy, limb-girdle MD, hypertrophic phenotypes like Danon disease, Friedreich ataxia, metabolic diseases such as fatty acid oxidation disorders, mitochondrial myopathies, myofibrillar myopathies, and channelopathies. Heart failure or arrhythmias may be the first presentation of MD [67]. In addition to genetics, elevated serum CK and lactic acid levels serve as key indicators for underlying MD. CMR helps assess cardiac involvement, monitor disease progression, and guide ICD therapy [68]. CMR can assess focal areas of fibrosis with LGE and interstitial abnormality with elevated T1 and ECV fraction.

## Future Directions and Conclusions

As mentioned, CMR can comprehensively evaluate many pathological features of cardiomyopathies, however, its widespread use is hindered by intricate protocols and the need for specialized expertise. Radiomics and artificial intelligence (AI) can address these challenges by improving the clinical efficiency and versatility of CMR. The integration of genetic testing with CMR is essential for understanding pathogenic pathways of inherited cardiomyopathies, such as inflammation and immunotherapy effects [24, 43]. Further research could involve AI integration of genetic data with CMR radiomic features, including perfusion data, to build a comprehensive database to further clarify the pathogenetics of cardiomyopathy associated with genetic abnormalities and identify specific therapeutic targets for personalized precision medicine.

## Key References

- Antonopoulos AS, Xintarakou A, Protonotarios A, Lazaros G, Miliou A, Tsioufis K, et al. Imagenetics for Precision Medicine in Dilated Cardiomyopathy. Vol. 17, Circulation: Genomic and Precision Medicine. 2024.
  - Cardiac MRI in dilated cardiomyopathy can detect key structural and functional changes in genotype-specific patterns, aiding in early diagnosis, risk stratification and potential precision medicine.
- Peretto G, De Luca G, Villatore A, Di Resta C, Sala S, Palmisano A, et al. Multimodal Detection and Targeting of Biopsy-Proven Myocardial Inflammation in Genetic Cardiomyopathies: A Pilot Report. JACC Basic Transl Sci. 2023;8(7).
  - CMR can detect myocardial inflammation in patients with genetic cardiomyopathies that may have a role in pathogenesis and target therapy.

## Data Availability

No datasets were generated or analysed during the current study.
